# New-Onset Systemic Lupus Erythematosus after mRNA SARS-CoV-2 Vaccination

**DOI:** 10.1155/2022/6436839

**Published:** 2022-02-11

**Authors:** Laisha Báez-Negrón, Luis M. Vilá

**Affiliations:** Division of Rheumatology, University of Puerto Rico, Medical Sciences Campus, San Juan, Puerto Rico

## Abstract

Systemic lupus erythematosus (SLE) is a multisystem autoimmune disease resulting from the interaction of genetic and environmental factors. In addition, some antiviral vaccines have been associated with the onset of SLE. Few cases of SLE occurring after SARS-CoV-2 mRNA have been reported. Herein, we report the case of a 27-year-old woman with type I diabetes mellitus and family history of SLE who presented with symmetric inflammatory polyarthritis of the proximal interphalangeal joints, metacarpophalangeal joints, wrists, knees, and ankles two weeks after receiving the second dose of the SARS-CoV-2 mRNA-1273 vaccine. Laboratory results revealed positive antinuclear, anti-dsDNA, anti-Ro, and anti-La/SSB antibodies and low C4 levels. She was initially treated with low-dose prednisone and hydroxychloroquine. Hydroxychloroquine was discontinued after she developed an urticarial rash. Subsequently, mycophenolate mofetil was added after she developed proteinuria. This case highlights the importance of considering the diagnosis of SLE in patients who present with inflammatory polyarthritis after COVID-19 vaccination.

## 1. Introduction

Systemic lupus erythematosus (SLE) is a chronic autoimmune disease characterized by dysregulation of the immune system [1]. Its etiopathogenesis involves the interaction of genetic and environmental factors such as ultraviolet light exposure, cigarette smoking, and infections [2]. Also, some vaccines have been linked with the onset of SLE including hepatitis B, human papilloma virus, and measles [3–6]. More recently, it has been reported that mRNA SARS-CoV-2 vaccines could trigger SLE flares and may induce the development of new-onset rheumatic diseases [7]. SARS-CoV-2 mRNA vaccines increase the levels of type I interferon (INF) which is not only known to play a critical role in the antiviral response but also an important cytokine in the pathogenesis of SLE [8–10]. Few cases of new-onset SLE occurring after SARS-CoV-2 mRNA vaccination have been reported [11, 12]. Herein, we describe a 27-year-old woman who developed SLE two weeks after receiving the second dose of the SARS-CoV-2 mRNA-1273 vaccine.

## 2. Case Presentation

A 27-year-old-woman with type 1 diabetes mellitus developed tiredness, weight loss (2 kg), symmetric polyarthralgia involving the proximal interphalangeal (PIP) joints, metacarpophalangeal (MCP) joints, wrists, knees, and ankles, and prolonged morning stiffness (1-2 hours) two weeks after receiving the second dose of SARS-CoV-2 mRNA-1273 vaccine. These symptoms were managed with nonsteroidal anti-inflammatory drugs, but she did not improve. She had no history of fever, anorexia, photosensitivity, malar rash, alopecia, mucosal ulcers, Raynaud's phenomenon, chest pain, cough, shortness of breath, or sicca symptoms. Her mother had SLE treated with hydroxychloroquine.

On initial visit, temperature was 37.3°C, blood pressure was 116/80 mmHg, and heart rate was 87 beats per minute. Physical examination showed symmetric joint tenderness and swelling of the 2^nd^ to 5^th^ PIPs and MCPs associated with limited range of movement and joint tenderness of the wrists, knees, and ankles bilaterally. The remainder of the physical exam was normal.

Laboratory tests revealed a white blood cell count of 7.6/*μ*L, hemoglobin of 13.9 g/dL, and platelet count of 372,000/*μ*L of. Serum creatinine was normal at 0.65 mg/dL. Alkaline phosphatase, aspartate aminotransferase, and alanine aminotransferase levels were normal. She had mild hypoalbuminemia at 3.3 g/dl. Urinalysis showed trace protein without microscopic hematuria, pyuria, or urinary casts. Spot urine protein to creatinine ratio was 0.47. Thyroid stimulating hormone and free thyroxine levels were normal. Levels of 25-hydroxyvitamin D were low at 21 ng/ml. Westergren erythrocyte sedimentation rate (ESR) was elevated at 88 mm/h, but C-reactive protein levels were normal. She had positive anti-nuclear (ANA, 1 : 160 titer, speckled pattern), anti-Ro, and anti-La antibodies. Anti-dsDNA antibodies were elevated at 46 IU/mL (normal range <10 IU/mL). She had low C4 levels at 12 mg/dL (normal range: 14–53 mg/dL) but had normal C3 levels. Anti-Smith and anti-RNP antibodies were negative. The lupus anticoagulant test, anti-cardiolipin (IgA, IgM, and IgG), and anti-B2 glycoprotein I (IgA, IgM, and IgG) antibodies were negative. Rapid plasma reagin (RPR), human immunodeficiency virus, hepatitis B, and hepatitis C tests were negative. X-rays of the hands and wrists were unremarkable.

Our patient fulfilled both the 2012 Systemic Lupus International Collaborating Clinics (total of 5 criteria; at least, 4 criteria are required for classification) and the 2019 European League Against Rheumatism/American College of Rheumatology (total of 19 points; at least, 10 points are required for classification) criteria for SLE [13, 14]. She was initially treated with hydroxychloroquine 300 mg daily and prednisone 5 mg daily. Two weeks after, she developed an urticarial rash. Hydroxychloroquine was discontinued with complete resolution of the rash. Over the next eight weeks, she persisted with inflammatory polyarthritis, proteinuria increased (spot urine protein to creatinine ratio = 0.64), ESR increased at 98 mm/h, and she continued with elevated anti-dsDNA antibodies (58 IU/mL) and low C4 levels (10 mg/dL). Prednisone dose was increased to 20 mg daily, and mycophenolate mofetil was started at 1 g daily. Four weeks later, polyarthritis subsided, but she continued with proteinuria (300 mg/dL) and elevated ESR at 86 mm/h. Mycophenolate mofetil dose was increased to 2 g daily, resulting in a favorable clinical response. Two months after this dose change, proteinuria decreased to 30 mg/dL. Prednisone dose could be lowered to 10 mg daily without her having a reactivation of her SLE.

## 3. Discussion

We describe a 27-year-old woman who developed SLE two weeks after receiving the second dose of the mRNA SARS-CoV-2 vaccine. SLE was manifested by constitutional symptoms, polyarthritis, subnephrotic-range proteinuria, low C4 levels, and positive ANA, and anti-ds-DNA antibodies, anti-SSA/Ro, and anti-SSB/La antibodies. Because our patient had a genetic predisposition for lupus, it is not surprising that she developed SLE after a potential trigger such as the COVID-19 vaccine. She has a first-degree relative with lupus and also has type I diabetes mellitus, both being strong risk factors for SLE [15–17]. Individuals who have a first degree relative with SLE have a 10 times increased risk of developing lupus [15]. Likewise, type 1 diabetes mellitus is strongly associated with SLE [16, 17]. The latter is not unexpected as type I diabetes mellitus and SLE share common pathophysiologic mechanisms including the association with PTPN22 and STAT4 polymorphisms [18].

To the best of our knowledge, two cases of new-onset SLE occurring after SARS-CoV-2 mRNA vaccination have been reported, both of which presented with cutaneous manifestations [11, 12]. Kreuter et al. reported a 79-year-old man who developed fatigue and papulosquamous and annular skin eruptions ten days after receiving the BNT162b2mRNA vaccine [11]. Laboratory tests disclosed positive for ANA (1 : 320 titer), rheumatoid factor, and anti-Ro and anti-La antibodies. Skin biopsy was consistent with subacute cutaneous lupus. Treatment with hydroxychloroquine and intravenous corticosteroids resulted in complete resolution of manifestations. Gambicher et al. described a 74-year-old woman who developed erythematous macules and papules. Laboratory tests showed positive ANA (1 : 640 titer, speckled pattern) and anti-Ro and anti-La antibodies. The patient was diagnosed with Rowell syndrome (erythema multiforme-like lesions coexisting with lupus) after a skin biopsy [12].

Like the abovementioned case reports, constitutional symptoms and ANA and anti-Ro and anti-La positivity occurred as the initial manifestations in our patient. Moreover, our patient developed subnephrotic-range proteinuria. Although she also had history of type I diabetes mellitus, she had no evidence of other microvascular organ damage such as peripheral neuropathy or retinopathy, which is usually concomitantly seen in patients with kidney involvement related to diabetes mellitus [19]. In addition, the time association between proteinuria and other SLE clinical manifestations as well as the presence of anti-dsDNA antibodies favors this manifestation as SLE related [20].

In addition to new-onset SLE after mRNA COVID-19 vaccination, two case reports have linked the SARS-CoV-2 viral vector vaccine with SLE [21, 22]. Zavala et al. reported a 23-year-old woman who developed alopecia, lymphopenia, nephrotic-range proteinuria, low C3 levels, positive ANA (1 : 1280 titer, homogenous pattern), and elevated anti-dsDNA antibodies one week after the first dose of the AZD1222 ChAdOX1 nCoV-19 vaccine. Kidney biopsy showed class V glomerulonephritis. She was treated with hydroxychloroquine, mycophenolate mofetil, and high-dose corticosteroids with improvement after three weeks of follow-up [21]. Patil et al. described a 22-year-old woman who experienced right knee pain two weeks after receiving the first dose of the Covishield vaccine. After the second dose, she developed fever, polyarthralgia, bipedal edema, and petechiae on lower extremities. Laboratories were remarkable for thrombocytopenia, albuminuria, positive ANA (1 : 320 titer), and elevated anti-dsDNA antibodies. She was treated with hydroxychloroquine, mycophenolate mofetil, and prednisolone. She had significant improvement of symptoms after one month [22].

SARS-CoV-2 mRNA vaccines may also induce exacerbations in previously controlled SLE patients. A recent study in a lupus cohort showed that 11.4% of patients had a disease exacerbation after vaccination [23]. The most common flare manifestations were oral ulcers, serositis, arthritis, pericarditis, thrombocytopenia, and renal involvement. Most patients did not require additional immunosuppressive treatment. Conversely, some case reports have documented SLE flares that have required immunosuppressive therapy [24, 25]. Niebel et al. reported a 73-year-old woman with subacute cutaneous lupus in full remission who experienced disseminated erythematous patches ten days after the first dose of the BNT16b2 mRNA vaccine. The symptoms improved with systemic and topical corticosteroids for three weeks [24]. Also, Tuschen et al. described a 41-year-old woman with lupus nephritis who was in clinical remission who developed nephrotic-range proteinuria seven days after receiving the first dose of the BNT 162b2 vaccine. The patient responded well to mycophenolate mofetil and prednisone therapy [25].

Based on the SARS-CoV-2 mRNA vaccine mechanism, it is not unforeseen that SARS-CoV-2 mRNA vaccines could cause new-onset SLE or induce lupus exacerbations. A lipid nanoparticle protects the mRNA and facilitates its transportation to the lymph nodes where it is engulfed by dendritic cells [8]. Once inside the cell, the mRNA is recognized in the endosome by toll-like receptors (TLRs) in dendritic cells [7, 26]. TLR-7 and TLR-8 bind single-stranded RNA, and once activated, it leads to the production of type I INF and proinflammatory cytokines [24]. Prior studies have demonstrated type I INF level elevation within 24 hours returning to normal within fourteen days after having administered SARS-CoV-2 mRNA vaccine [27]. On the other hand, SLE is characterized by impaired phagocytic clearance of apoptotic material and immune tolerance disruption to self-antigens. The immune complexes composed of autoantibodies and apoptotic debri are endocytosed and sensed by TLRs [28]. Since SLE complexes are made of endogenous RNA and DNA nucleic acids, they are detected by TLR-7 and TLR-9 [28, 29]. These pattern recognition receptors create a signal leading to the expression of IL-6, TNF-alpha, and type I INF comparable to the effect of COVID-19 mRNA vaccines [28–30].

Antiviral vaccines, other than COVID-19, such as hepatitis B, human papilloma virus, and measles vaccines have been linked with SLE [3–6]. In a murine model of lupus, hepatitis B vaccine and its adjuvants accelerate SLE by producing glomerulonephritis and elevated levels of anti-dsDNA antibodies [6]. Adjuvants can stimulate an immune response by acting as a ligand for TLRs [31]. In contrast to the SARS-CoV-2 vaccine, HPV vaccine is thought to cause SLE through molecular mimicry [32]. As noted for our patient, it is proposed that these antiviral vaccines could induce lupus in genetically predisposed individuals [31, 32].

Although mRNA SARS-CoV-2 vaccines contain no adjuvants, it is important to highlight that non-mRNA vaccines may contain adjuvants that could induce an inflammatory response [33]. The autoimmune/inflammatory syndrome disease induced by adjuvants (ASIA) or Shoenfeld's syndrome is characterized by constitutional symptoms, arthralgias, myalgias, myositis, neurological manifestations, and the appearance of autoantibodies [34]. Some adjuvants induce this inflammatory response through the expression of proinflammatory cytokines such as interferon, IL-1, and IL-6 [35]. Furthermore, ASIA syndrome has been linked with the occurrence of SLE and antiphospholipid syndrome, among other autoimmune disorders [35]. It appears that this syndrome occurs more frequently in patients with genetic predisposition to develop autoimmunity.

In summary, we report a woman who developed SLE after mRNA SARS-CoV-2 vaccination. Although it is unusual, it is important to maintain a high level of awareness of SLE in patients who present with inflammatory polyarthritis after mRNA SARS-CoV-2 vaccination, particularly in those with risk factors for autoimmune diseases. Early recognition is critical to initiate prompt and effective therapy.

## Data Availability

Data can be obtained from the corresponding author upon request.

## References

[1] Tsokos G. C. (2020). Autoimmunity and organ damage in systemic lupus erythematosus. *Nature Immunology*.

[2] Parks C. G., de Souza Espindola Santos A., Barbhaiya M., Costenbader K. H. (2017). Understanding the role of environmental factors in the development of systemic lupus erythematosus. *Best Practice & Research Clinical Rheumatology*.

[3] Soldevilla H., Briones S., Navarra S. (2012). Systemic lupus erythematosus following HPV immunization or infection?. *Lupus*.

[4] de Mattos A. B. N., Garbo Baroni L., Zanotto L. d. L. (2021). Subacute cutaneous lupus erythematosus triggered after measles vaccination. *Lupus*.

[5] Agmon-Levin N., Zafrir Y., Paz Z., Shilton T., Zandman-Goddard G., Shoenfeld Y. (2009). Ten cases of systemic lupus erythematosus related to hepatitis B vaccine. *Lupus*.

[6] Agmon-Levin N., Arango M. T., Kivity S. (2014). Immunization with hepatitis B vaccine accelerates SLE-like disease in a murine model. *Journal of Autoimmunity*.

[7] Watad A., De Marco G., Mahajna H. (2021). Immune-mediated disease flares or new-onset disease in 27 subjects following mRNA/DNA SARS-CoV-2 vaccination. *Vaccines*.

[8] Teijaro J. R., Farber D. L. (2021). COVID-19 vaccines: modes of immune activation and future challenges. *Nature Reviews Immunology*.

[9] Stegelmeier A. A., Darzianiazizi M., Hanada K. (2021). Type I interferon-mediated regulation of antiviral capabilities of neutrophils. *International Journal of Molecular Sciences*.

[10] Tang W., Askanase A. D., Khalili L., Merrill J. T. (2021). SARS-CoV-2 vaccines in patients with SLE. *Lupus Science & Medicine*.

[11] Kreuter A., Licciardi-Fernandez M. J., Burmann S. N., Burkert B., Oellig F., Michalowitz A.-L. (2022). Induction and exacerbation of subacute cutaneous lupus erythematosus following mRNA-based or adenoviral vector-based SARS-CoV-2 vaccination. *Clinical and Experimental Dermatology*.

[12] Gambichler T., Scholl L., Dickel H., Ocker L., Stranzenbach R. (2021). Prompt onset of Rowell’s syndrome following the first BNT162b2 SARS-CoV-2 vaccination. *Journal of the European Academy of Dermatology and Venereology*.

[13] Petri M., Orbai A. M., Alarcón G. S. (2012). Derivation and validation of the Systemic Lupus International Collaborating Clinics classification criteria for systemic lupus erythematosus. *Arthritis & Rheumatism*.

[14] Aringer M., Costenbader K., Daikh D. (2019). 2019 European League against rheumatism/American College of Rheumatology classification criteria for systemic lupus erythematosus. *Arthritis & Rheumatology*.

[15] Ulff-Møller C. J., Simonsen J., Kyvik K. O., Jacobsen S., Frisch M. (2017). Family history of systemic lupus erythematosus and risk of autoimmune disease: nationwide Cohort Study in Denmark 1977–2013. *Rheumatology*.

[16] Hemminki K., Li X., Sundquist J., Sundquist K. (2009). Familial association between type 1 diabetes and other autoimmune and related diseases. *Diabetologia*.

[17] Bao Y. K., Weide L. G., Ganesan V. C. (2019). High prevalence of comorbid autoimmune diseases in adults with type 1 diabetes from the HealthFacts database. *Journal of Diabetes*.

[18] Zervou M. I., Mamoulakis D., Panierakis C., Boumpas D. T., Goulielmos G. N. (2008). STAT4: a risk factor for type 1 diabetes?. *Human Immunology*.

[19] Avogaro A., Fadini G. P. (2019). Microvascular complications in diabetes: a growing concern for cardiologists. *International Journal of Cardiology*.

[20] Goilav B., Putterman C. (2015). The role of anti-DNA antibodies in the development of lupus nephritis: a complementary, or alternative, viewpoint?. *Seminars in Nephrology*.

[21] Zavala-Miranda M. F., González-Ibarra S. G., Pérez-Arias A. A., Uribe-Uribe N. O., Mejia-Vilet J. M. (2021). New-onset systemic lupus erythematosus beginning as class V lupus nephritis after COVID-19 vaccination. *Kidney International*.

[22] Patil S., Patil A. (2021). Systemic lupus erythematosus after COVID-19 vaccination: a case report. *Journal of Cosmetic Dermatology*.

[23] Izmirly P. M., Kim M. Y., Samanovic M. (2022). Evaluation of immune response and disease status in SLE patients following SARS-CoV-2 vaccination. *Arthritis & Rheumatology*.

[24] Niebel D., Ralser-Isselstein V., Jaschke K., Braegelmann C., Bieber T., Wenzel J. (2021). Exacerbation of subacute cutaneous lupus erythematosus following vaccination with BNT162b2 mRNA vaccine. *Dermatologic Therapy*.

[25] Tuschen K., Bräsen J. H., Schmitz J., Vischedyk M., Weidemann A. (2021). Relapse of class V lupus nephritis after vaccination with COVID-19 mRNA vaccine. *Kidney International*.

[26] Linares-Fernández S., Lacroix C., Exposito J. Y., Verrier B. (2020). Tailoring mRNA vaccine to balance innate/adaptive immune response. *Trends in Molecular Medicine*.

[27] Ntouros P. A., Vlachogiannis N. I., Pappa M. (2021). Effective DNA damage response after acute but not chronic immune challenge: SARS-CoV-2 vaccine versus Systemic Lupus Erythematosus. *Clinical Immunology*.

[28] Kim J. M., Park S. H., Kim H. Y., Kwok S.-K. (2015). A plasmacytoid dendritic cells-type I interferon Axis is critically implicated in the pathogenesis of systemic lupus erythematosus. *International Journal of Molecular Sciences*.

[29] Bonegio R. G., Lin J. D., Beaudette-Zlatanova B., York M. R., Menn-Josephy H., Yasuda K. (2019). Lupus-associated immune complexes activate human neutrophils in an Fc*γ*RIIA-dependent but TLR-independent response. *The Journal of Immunology*.

[30] Lorenz G., Anders H. J. (2015). Neutrophils, dendritic cells, toll-like receptors, and interferon-*α* in lupus nephritis. *Seminars in Nephrology*.

[31] Bragazzi N. L., Watad A., Sharif K. (2017). Advances in our understanding of immunization and vaccines for patients with systemic lupus erythematosus. *Expert Review of Clinical Immunology*.

[32] Segal Y., Shoenfeld Y. (2018). Vaccine-induced autoimmunity: the role of molecular mimicry and immune crossreaction. *Cellular and Molecular Immunology*.

[33] Park K. S., Sun X., Aikins M. E., Moon J. J. (2021). Non-viral COVID-19 vaccine delivery systems. *Advanced Drug Delivery Reviews*.

[34] Watad A., Quaresma M., Bragazzi N. L. (2018). The autoimmune/inflammatory syndrome induced by adjuvants (ASIA)/Shoenfeld’s syndrome: descriptive analysis of 300 patients from the international ASIA syndrome registry. *Clinical Rheumatology*.

[35] Watad A., Quaresma M., Brown S. (2017). Autoimmune/inflammatory syndrome induced by adjuvants (Shoenfeld’s syndrome)-an update. *Lupus*.

